# DUSP5 Downregulation in Nucleus Accumbens Core Correlates with Cocaine-Induced Maladaptive Synaptic Plasticity

**DOI:** 10.3390/cells15010032

**Published:** 2025-12-23

**Authors:** Juan Pablo Taborda-Bejarano, Michael Meyerink, Debbie C. Crans, Ramani Ramchandran, Constanza Garcia-Keller

**Affiliations:** 1 Department of Pharmacology and Toxicology, Medical College of Wisconsin, 8701 Watertown Plank Road, Milwaukee, WI 53226, USA; jtaborda@mcw.edu (J.P.T.-B.); mey171@osumc.edu (M.M.); 2 Department of Chemistry and Cell and Molecular Biology Program, Colorado State University, Fort Collins, CO 90523, USA; 3 Department of Pediatrics, Division of Neonatology, Children’s Research Institute (CRI) Developmental Vascular Biology Program, Translational and Biomedical Research Center, 8701 Watertown Plank Road, Milwaukee, WI 53226, USA

**Keywords:** DUSP5, pERK, phosphatase, cocaine, reinstatement

## Abstract

The United States is currently facing a drug overdose epidemic. The nucleus accumbens core (NAcore), a brain region critical for reward and aversion behaviors, undergoes structural and functional synaptic adaptations in response to chronic drug exposure. However, the molecular mechanisms underlying these adaptations remain poorly understood. In this study, we investigate the role of dual-specificity phosphatase 5 (DUSP5), a phosphatase known to deactivate extracellular signal-regulated kinase (ERK), in cocaine-induced neuroplasticity. While prior research has linked other DUSP family members to various drugs of abuse, the specific role of DUSP5 in cocaine addiction remains unexplored. We hypothesized that lack of DUSP5 contributes to cocaine-induced maladaptive synaptic plasticity in NAcore. To test this, we employed a rat cocaine self-administration model and molecular analyses and mined publicly available single-cell RNA sequencing data from cocaine-treated NAcore. Our findings reveal a role for DUSP5 in cocaine-related synaptic and behavioral adaptations, highlighting DUSP5 and DUSP5-associated signaling pathways as potential mechanisms underlying substance use disorders and as candidates for therapeutic intervention.

## 1. Introduction

Cocaine addiction continues to pose a major public health challenge in the United States. In 2021, an estimated 1.4 million individuals met the criteria for cocaine use disorder. Alarmingly, the rate of cocaine-related overdoses is increasing, largely due to the combined use of cocaine with synthetic opioids like fentanyl. This pattern of polysubstance abuse marks the so-called “fourth wave” of the opioid crisis, defined by the convergence of stimulant and opioid use [1,2]. The rising prevalence of stimulant use has introduced additional complications, including adverse behavioral outcomes such as neurological impairments, suicidal ideation, psychosis, aggression, and violence, while also complicating treatment approaches [1,2,3].

Clinical evidence underscores the need for a deeper understanding of how drugs alter brain function to effectively treat substance use disorder (SUD) [4]. SUD is closely linked to synaptic changes within the nucleus accumbens (NAc), a brain region central to reward processing, motivation, and addiction [5]. The NAc, or ventral striatum, contains core and shell subregions that play distinct roles in addiction-related behaviors [6]. The core is primarily associated with driving goal-directed actions and drug-seeking (reinstatement) behavior whereas the shell is involved in motivational processes and the formation of stimulus-reward associations [6,7]. Within the NAcore, approximately 90% of neurons are medium spiny neurons (MSNs) that express either D1 or D2 dopamine receptors [8]. Activation of D1-MSNs has been shown to facilitate motivated behaviors [9], whereas D2-MSN activation tends to suppress them. Long-lasting, experience-dependent changes in synaptic strength within the NAcore are believed to underlie drug-induced neuroplasticity [7,10]. This plasticity is evident both structurally, through changes in dendritic morphology, and functionally, via altered glutamatergic receptor signaling, both of which influence one another [11,12]. Despite significant efforts, therapeutic strategies aimed at reversing drug-induced synaptic plasticity have yielded limited success. This is due in part to an incomplete understanding of the mechanisms governing synaptic plasticity under both normal and drug-altered conditions. In the present study, we address this gap by investigating a novel molecular target, the dual-specificity phosphatases (DUSPs), with a particular focus on DUSP5, a member of this family, and its role in SUD.

DUSPs are dual-specificity phosphatases in that they remove phosphate groups (dephosphorylate) from serine (S), threonine (T) and tyrosine (Y) residues. Specifically, DUSP5 inactivates extracellular regulated kinase (ERK) by removing phosphate groups both on phosphorylated threonine and tyrosine (pT and pY) residues [13,14,15]. *DUSP5* and *DUSP6* mRNA expression is upregulated (~1.4-fold) in the prefrontal cortex following heroin abstinence [16]. Conversely, *DUSP5* expression in the NAc decreases (~0.67-fold) shortly after heroin injection [17]. Methamphetamine exposure also affects DUSP expression: *DUSP1* and *DUSP6* mRNAs and protein levels were increased in multiple brain regions, including the cortex, striatum, thalamus, and hippocampus right after a single injection [18,19]. Another DUSP family member implicated in SUD is DUSP15. *DUSP15* mRNA and protein expression were downregulated in NAc after 24 h and 10 days after the last morphine injection [20]. DUSP15 over-expression using a virus prevented the morphine-paired contextual memory, facilitated extinction, and both inhibited reinstatement and abolished ERK activation [20]. Additionally, a variant of the DUSP27 gene (rs950302) is associated with heroin addiction in African Americans [21]. Collectively, these findings suggest that DUSP genes may contribute to both the vulnerability and progression of addictive behaviors, potentially playing key roles in relapse by influencing drug-seeking behavior regarding psychostimulants and opioids.

Our team has been investigating DUSP5 in the vascular context and identified a novel molecular mechanism in its regulation of p-ERK [15,22,23]. Previous studies [24,25] have shown that DUSP5 is essential for regulating hippocampal dentate gyrus neuroplasticity, a brain region important for learning and memory. Based on these findings, we hypothesized that DUSP5 may contribute to maladaptive synaptic plasticity in the NAcore. To test this hypothesis, we employed a multidisciplinary approach combining molecular and behavioral methods. These methods included performing single-cell RNA sequencing analysis on publicly available data from the NAcore tissue of cocaine-treated rats, conducting standard rat cocaine self-administration, analyzing dendritic spine morphology, and performing correlation analyses between behavior and structural synaptic plasticity.

## 2. Materials and Methods

### 2.1. Animal Housing and Surgery

Nine male Long–Evans rats were individually housed using a 12/12 h light/dark cycle with ad libitum food and water in a temperature and humidity-controlled environment. All animals were allowed to acclimate to the vivarium environment for a week before surgery and experimentation occurred during the dark phase. Rats were ~70 days old when they were anesthetized with isoflurane and implanted with indwelling jugular catheters (surgical details have been described previously [26,27]) and received ketorolac as a preoperative analgesic. All procedures were in accordance with the National Institutes of Health Guide for the Care and Use of Laboratory Animals and the Assessment and Accreditation of Laboratory Animal Care (IACUC). Our animal protocol at Medical College of Wisconsin is ID: AUA00007874, first approved on 29 September 2022, renewed in September 2025.

### 2.2. Drugs and Reagents Used

Cocaine hydrochloride was supplied by the National Institute of Drug Abuse. NAcore MSNs were labeled with a virus (0.75 µL/hemisphere at a rate of 0.15 mL/min) that drove expression of membrane targeted mCherry under control of the synapsin promoter (pAAV2-shRNA, titer 1.39 × 10^15^ GC/mL). A custom made AAV-shRNA vector consisted of a CMV promoter driving mCherry with an SV40 polyadenylation signal followed downstream by a U6 polymerase III promoter and polymerase III termination signal [28]. Rabbit anti-DUSP5 monoclonal antibody (Abcam, Waltham, MA, USA; Cat # ab200708, 1:500) and amplification of the viral reporter was performed at the same time using anti-mCherry (Polyclonal Chicken anti-Discosoma mCherry Antibody (IF, WB) LS-C204825, 1:500) host in chicken. Secondary antibodies—anti-chicken and anti-rabbit—Alexa Fluor-conjugated to the appropriate species were used as well (Life Technologies based in Carlsbad, CA, USA, 1:1000).

### 2.3. Self-Administration (SA), Extinction, and Reinstatement

All procedures occurred in standard operant chambers equipped with two retractable levers, a house light, cue light, and 2900-Hz tone generator (Med Associates based in Fairfax/St. Albans, VT, USA). Five male cocaine-treated and 4 male yoked-saline rats were used in this study. Before cocaine or saline SA training, animals were food deprived for 48 h and then underwent a single 2-h food training session in which presses on the active lever resulted in the delivery of a single food pellet (45 mg, Noyes) on a fixed-ratio 1 (FR1) schedule of reinforcement. Following food training, animals were left with food ad libitum for the remainder of the experiments. One day later, animals began 2-h sessions of cocaine SA on an FR1 schedule with a 20-s time out. Each active lever press resulted in an infusion of cocaine hydrochloride (0.25 mg/infusion, volume is 50 μL) and simultaneously triggered a compound cue, consisting of a light positioned above the active lever and a 2900 Hz tone, serving as a conditioning stimulus. An inactive lever was also provided to control for non-motivated responses Active lever presses made during the time out were counted but did not result in drug delivery. Rats underwent SA for a minimum of 10 days, until they met maintenance criteria of ≥10 infusions of cocaine over 10 days, as well as discrimination between active and inactive levers (>75% presses on active lever). A subset of animals served as yoked-saline controls and received a noncontingent saline infusion paired with light and tone cues according to a pre-programmed pattern of responding based on the cocaine average but were otherwise treated identically throughout.

Following successful acquisition and maintenance of cocaine SA, extinction training (2 h/d) began. During extinction, presses on the previously active lever were recorded but no longer produced drug or presentation of the drug-paired cues. All rats underwent at least 8 days of extinction, or until two consecutive days revealed ≤25 active lever presses.

Cue-induced drug reinstatement training was conducted following extinction. During reinstatement sessions, animals were tested under a FR1 schedule, in which each active lever press resulted in presentation of the previously drug-paired light/tone cues used during SA, but without cocaine delivery. To initiate the session and signal cue availability, a single non-contingent (“free”) cue presentation was delivered at the start of the session. Thereafter, cue presentations occurred only in response to active lever presses. For dendritic spine analysis, both saline- and cocaine-exposed animals were removed from the operant chambers and euthanized 15 min after the start of the reinstatement session. This time point was chosen to capture rapid, cue-evoked neuroplastic events that occur immediately upon re-exposure to drug-associated cues. Behavioral analysis in our cohort, consistent with prior reports, shows that the first 15 min of reinstatement correspond to the peak in cue-induced lever pressing, during which animals exhibit their highest levels of drug seeking. All behavioral sessions lasted 2 h and were performed at the same time each day.

### 2.4. Quantification of Dendritic Spine Morphology and Immunohistochemistry

Rats were anesthetized with isoflurane as per our approved animal protocol. Transcardial perfusions was performed with PBS followed by fixing the tissue in 4% paraformaldehyde (PFA) in PBS. Brains were removed and placed in sucrose buffer for 24 h, then coronally sectioned at 100 μm in PBS on a vibratome. For immunohistochemical detection of DUSP5, sections were incubated with a rabbit recombinant primary antibody against DUSP5 (1:500) for 48 h at 4 °C with gentle agitation (as described in [27]). The mCherry viral reporter signal was amplified using anti-mCherry antibody. Following incubation with primary antibodies, sections were washed and followed by an overnight incubation at 4°C with appropriate species-specific Alexa Fluor-conjugated secondary antibody (1:1000, Life Technologies). After a brief PBS wash, tissue samples were placed onto slides in an aqueous medium and mounted with ProLong gold Antifade (Life Technologies) to preserve fluorescence intensity over time.

Spine morphology was quantified as described in detail previously [26]. Briefly, images of MSN-labeled segments were taken on a confocal microscope (Leica SP8 based in Germany) using a 561-nm laser line. Images of dendrites were taken through a 63× oil immersion objective with a numerical aperture of 1.4, using a 3.5× digital zoom. Images were deconvolved via Leica LASX imaging (version number 3.5.7.23225) software before analysis, and a 3-D perspective was rendered by the Surpass module of Imaris software package version 9.9 (Bitplane based in the United Kingdom, version number 11.0). Final data set voxel dimensions were of 0.049 μm in the *xy* plane and 0.3 μm in the *z* plane. The smallest quantifiable diameter spine head was set to 0.14 μm. Only spines on dendrites beginning >75 μm and ending <200 μm distal to the soma and after the first branch point were quantified on cells localized to the NAcore. The length of quantified segments was 45–55 μm. DUSP5 protein immunoreactivity was quantified in the same dendritic segments used for morphological analyses. To assess colocalization of DUSP5 with virally labeled dendrites, the total volume (µm^3^) of DUSP5 puncta signal was measured and normalized to the volume (µm^3^) of the corresponding dendritic shaft or spine, yielding an index of DUSP5 signal intensity within the isolated dendritic compartments (see Supplemental Figure S1 for details). All imaging and subsequent analyses were conducted blinded to experimental group assignments.

### 2.5. Statistics

All statistical analyses were performed using GraphPad Prism version 10. All measurements were conducted by experimenters blinded to treatment conditions. For drug SA, acquisition was defined as achieving a criterion of ≥10 infusions per 120-min session across two consecutive days. Self-administration data were analyzed using a two-way ANOVA with repeated measures across time. Cocaine and saline groups were evaluated separately, as were self-administration and extinction phases. Cue-induced reinstatement was also analyzed using two-way ANOVA. Dendritic spine morphology was analyzed using Imaris (Bitplane), including measurements of medium spiny neuron (MSN) dendritic spine density, spine head diameter, and DUSP5 immunoreactivity in both the spine and shaft compartments. For dendritic spine and DUSP5 immunoreactivity analyses, data normality was assessed using the D’Agostino & Pearson normality test. Comparisons between groups were made using t test Nested ANOVA. Correlation analyses and linear regressions were performed using GraphPad Prism based in San Diego, CA, USA.

### 2.6. Methods of Phillips and Tuscher et al., 2023 [29]

This study examined cocaine-induced transcriptional and cellular adaptations in the rat nucleus accumbens using behavioral assays, snRNA-seq, cross-species transcriptomic comparisons, and RNAscope-based histology. Full methodological details are available in the reported manuscript; a summary is provided below:

#### 2.6.1. Behavioral Procedures

Adult male and female rats received intraperitoneal injections of cocaine (20 mg/kg) or saline. Animals were exposed either to a single acute dose or to repeated daily injections for seven days.

#### 2.6.2. Single-Nucleus Isolation and snRNA-Seq

NAc tissue from four animals per condition (matched by sex, treatment, and exposure duration) was pooled for single-nucleus dissociation. Nuclei were captured using 10× Genomics Chromium Single Cell 3′ v3 chemistry (based in Pleasanton, CA, USA). Acute cocaine samples yielded 15,655 nuclei, and repeated cocaine samples yielded 23,599 nuclei, sequenced on an Illumina NextSeq 500 based in San Diego, CA, USA. Raw reads were aligned to the rat mRatBn7.2 (Rn7) genome using Cell Ranger. Subsequent processing was conducted in Seurat, including quality filtering, log-normalization, integration of GEM wells, dimensionality reduction, and clustering. Ambient RNA contamination was removed with SoupX, and heterotypic doublets were identified and excluded (repeated exposure dataset only) using DoubletFinder v2.0.3.

#### 2.6.3. Differential Expression and Functional Analyses

To enable statistically robust comparisons, counts were pseudobulked by GEM v1.0 well within each cell type, and genes with fewer than five counts were excluded. Differential expression analyses were performed with DESeq2 v1.28.1 using the likelihood ratio test, incorporating dataset, treatment, and sex as covariates where appropriate.

Additional downstream analyses included:

- Gini coefficient calculations to assess transcriptional specificity.
- Rank–rank hypergeometric overlap (RRHO) to quantify transcriptomic similarity across conditions.
- Gene ontology analyses via g:Profiler using Benjamini–Hochberg correction.

All analytical code is publicly available on the project GitHub repository.

#### 2.6.4. Cross-Species Conservation Analysis

To compare gene-expression signatures across mammalian species, pseudobulk DEGs were used to compute t-statistics for each gene within MSN subtypes. The t-statistic incorporated mean expression differences, pooled variance, and nucleus number per cell type. Pearson correlations between species were then calculated in R version 4.5.

#### 2.6.5. Tissue Preparation and RNAscope Fluorescent In Situ Hybridization

Brains from drug-naïve rats were flash-frozen, sectioned coronally (10 μm), and processed using the RNAscope Multiplex Fluorescent v2 assay. Probes targeting Ebf1, Htr4, and Drd1a were applied, and fluorophores were visualized using a Keyence BZ-X800 microscope at 20× and 100× magnification.

#### 2.6.6. Image Analysis

Stitched images were analyzed in ImageJ V2.9.0/1.53t. NAc nuclei were segmented using StarDist based on DAPI staining, and these ROIs were used to quantify normalized fluorescence intensities for each probe. This enabled classification of *Drd1a+*, *Drd1a+/Ebf1+*, *Drd1a+/Htr4+*, and triple-positive cell populations.

## 3. Results

### 3.1. DUSPs mRNA Levels Changes in D1-MSN in NAcore Isolated from Non-Contingent Saline- and Cocaine-Injected Rats

**Figure 1:**
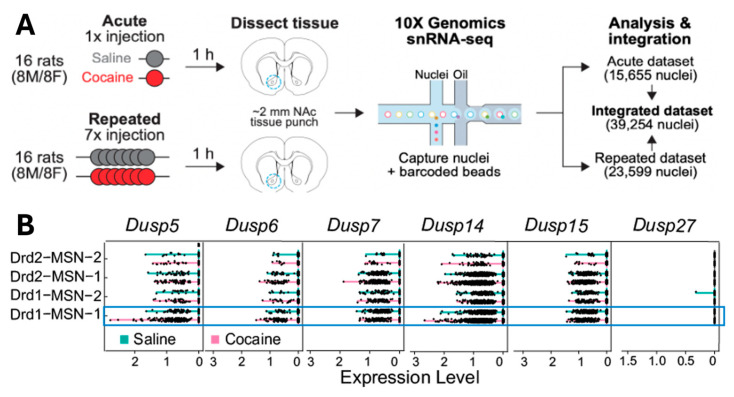
DUSP5 mRNA is selectively upregulated in D1-MSNs of the NAc following acute cocaine exposure. (**A**): Schematic of the experimental design from a publicly available single-nucleus RNA-sequencing (snRNA-seq) dataset [29]. Rats were treated with either acute (1) or repeated (7) intraperitoneal (i.p.) injections of cocaine or saline. One hour after the final injection, ~2 mm tissue punches from the NAc were collected for snRNA-seq using the 10× Genomics platform. The integrated dataset contains transcriptomic profiles from 39,254 nuclei, with data stratified by cell type. (**B**): Gene expression levels (log-transformed) for Dusp5, Dusp6, Dusp7, Dusp14, Dusp15, and Dusp27 across distinct brain cell populations, including Drd1- and Drd2-expressing MSN, interneurons, glial cells, and astrocytes. Pink lines represent expression following cocaine, and teal lines represent saline controls. Notably, Dusp5 mRNA is selectively upregulated in Drd1-MSNs (D1-MSNs) following acute cocaine administration, while other DUSPs show no consistent cocaine-related changes across cell types. This Dusp5-specific induction in D1-MSNs highlights its potential role in mediating early, cell-type-specific transcriptional responses to cocaine exposure in the NAc.

To examine how cocaine exposure affects DUSP family gene expression across different cell types in the NAc, we analyzed a publicly available single-nucleus RNA sequencing (snRNA-seq) dataset from rats treated with acute (1 injection) and repeated (7 injections) intraperitoneal cocaine [29]. NAc tissue was collected 1 h after the final injection, and nuclei were isolated for transcriptomic profiling (Figure 1A). Data from acute and repeated treatment groups were integrated to examine cocaine-induced changes in mRNA expression across 16 annotated brain cell populations, including D1- and D2-expressing MSNs, interneurons, astrocytes, oligodendrocytes, and microglia. Among the DUSP family members examined (Dusp5, Dusp6, Dusp7, Dusp14, Dusp15, and Dusp27), only Dusp5 showed a selective increase in expression in D1-MSNs within the NAcore (Drd1-MSN-1), but not in NAc shell (Drd1-MSN-2), following cocaine treatment (Figure 1B; blue box and Table 1). The difference between Drd1-MSN-1 and Drd1-MSN-2, as described in [29] lies in the varying expression levels of *Htr4* (which encodes the serotonin receptor 4) and *Calb1* (which encodes the calcium-binding protein Calbindin 1). These genes are enriched in the NAcore, indicating that the two Drd1-expressing MSN subtypes identified through snRNA-seq are distinct both transcriptionally and spatially. No changes in *Dusp5* expression were observed in D2-MSNs from either the NAc core or shell (Drd2-MSN-1 or Drd2-MSN-2), nor in other neuronal or glial cell types. Additionally, expression of the other DUSP genes remained largely unchanged across all cell populations analyzed (Supplemental Figure S2 and Table 1). These results suggest that *Dusp5* mRNA is selectively upregulated in D1-MSNs of the NAcore following cocaine exposure, identifying DUSP5 as a potential cell-type-specific regulator of cocaine-induced neuroadaptations.

### 3.2. Cocaine-Treatment Decreased DUSP5 Immunoreactivity in NAcore MSNs

**Figure 2:**
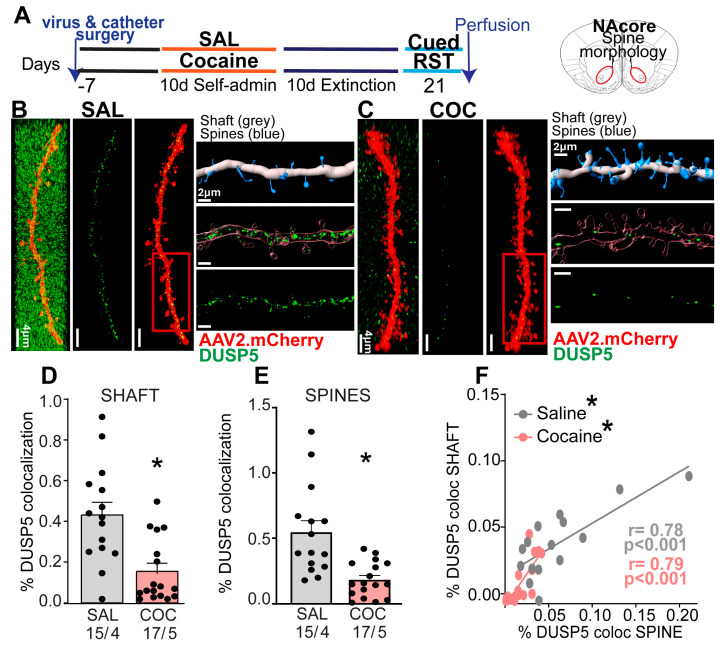
Reduced DUSP5 Protein Levels in NAcore MSNs During Cue-Evoked Cocaine Seeking. (**A**) Experimental timeline illustrating the sequence of viral and catheter surgeries, SA training, and the time of perfusion for both cocaine (COC) and saline (SAL) groups. (**B**,**C**) Representative dendritic segments from NAcore MSNs labeled with AAV2-shRNA-mCherry (red) and DUSP5 (green) in SAL and COC groups. The three vertical panels show confocal images of DUSP5 puncta (green) and dendritic labeling (red): **left**—merged full image, **middle**—isolated dendrite, **right**—DUSP5 puncta localized within the dendrite. Scale bar = 4 µm. Next to it, horizontal panels display high-magnification insets of each segment. Also shown is the quantification method: **top**—3D Imaris Bitplane reconstruction distinguishing shaft (gray) and spines (blue); **middle**—isolated dendritic segment with DUSP5 puncta; **bottom**—quantification of DUSP5 colocalization in shaft and spines. (**D**) Significant reduction of DUSP5 immunoreactivity in dendritic shafts of COC group compared to SAL group (t test Nested Anova F (1,7) = 6.23, *p* = 0.04). (**E**) Significant reduction of DUSP5 immunoreactivity in dendritic spines of COC group compared to SAL group (t test Nested Anova F (1,7) = 7.361, *p* = 0.03). (**F**) Positive correlation between DUSP5 immunoreactivity in dendritic shafts and spines in both SAL and COC groups (saline: r = 0.78, *p* < 0.001; cocaine: r = 0.79, *p* < 0.001). Data are shown as mean ± SEM. *N* shown as number of neurons quantified over number of animals in each condition; * *p* < 0.05 comparing COC to SAL.

To determine whether DUSP5 expression changes observed in data analysis of snRNA-seq, we assessed DUSP5 expression at the protein level in a self administration cocaine rat model. We implanted an intravenous catheter to allow SA of cocaine and microinjected AAV2-shRNA-mCherry to label dendrites for morphologic and immunoreactivity analysis of DUSP5 (Figure 2A). Rats were trained to self-administer cocaine along with saline controls (Supplemental Figure S3A). No statistical difference was observed between total number of saline infusions (flat rate) compared to cocaine-treated rats (Supplemental Figure S3B). After animals completed extinction training, reinstated cocaine seeking was induced for 15 min by restoring cocaine-paired cues (Supplemental Figure S3C). We and others have shown that this SA protocol induces synaptic plasticity in NAcore MSNs [10,26,27,30], providing the rationale for using this model in our study.

Brain slices were prepared of NAcore and stained for DUSP5 (Figure 2B,C). We quantified the DUSP5 immunostaining results using a 3D-rendering method previously published by our group [27] and described Supplemental Figure S1. We found decreased DUSP5 immunoreactive puncta within NAcore dendrites after 15 min of cue-induced reinstatement in cocaine-treated animals, compared with levels of immunoreactive DUSP5 puncta from saline-treated rats. DUSP5 immunoreactivity was significantly reduced in shaft and spines after cocaine treatment (Figure 2D,E). Furthermore, we observed a positive correlation between DUSP5 levels in shafts and spines across both treatment groups (Figure 2F). These findings demonstrate that DUSP5 protein levels are diminished in both dendritic shafts and spines of NAcore MSNs during cue-induced cocaine reinstatement and suggest a coordinated regulation of DUSP5 immunoreactivity within these subcellular compartments.

### 3.3. DUSP5 Immunoreactivity in NAcore MSN Correlates with Active Lever Presses During Reinstatement

**Figure 3:**
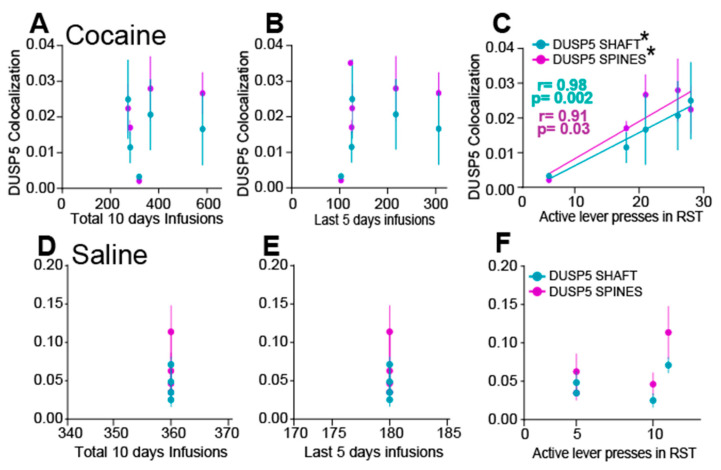
DUSP5 Immunoreactivity positively correlates with active lever pressing during cue-induced reinstatement. (**A**,**B**) No significant correlation was observed between DUSP5 immunoreactivity in dendritic shafts or spines with (**A**) total cocaine infusions or (**B**) cocaine infusions during the last 5 days of SA. (**C**) A significant positive correlation was found between DUSP5 immunoreactivity in spine heads and the number of lever presses during cue-induced reinstatement (spines: r = 0.91, *p* = 0.03; shaft: r = 0.98, *p* = 0.002). (**D**,**E**) Replication of analyses in a saline cohort showed no correlation between DUSP5 immunoreactivity in shaft or spine heads and (**D**) total infusions or (**E**) infusions during the last 5 days. (**F**) No correlation between DUSP5 levels in spine heads and lever pressing during reinstatement. This figure illustrates the correlations between total infusions, infusions during the last five days, or active lever presses and the mean ± SEM of DUSP colocalization within dendritic shafts or spines. * *p* < 0.05 corresponds to positive regression line.

To support a causal relationship between immunoreactivity of DUSP5 in shaft and spines and behavior we performed correlation analysis. Specifically, we examined the association between DUSP5 inmunoreactive puncta and (1) total number of infusions, (2) infusions during the last five days of SA, and (3) active lever presses during cue-induced reinstatement. Simple linear regression revealed no significant correlations between DUSP5 immunoreactivity (in either spines or shafts) and total cocaine infusions (Figure 3A), saline infusions (Figure 3D), or infusions during the final five days of SA (Figure 3B,E). However, a significant positive correlation was observed between DUSP5 immunoreactivity in dendritic spines and shaft with the number of active lever presses during reinstatement in cocaine-treated animals (Figure 3C). No such correlation was found in saline-treated animals (Figure 3F). These results suggest that DUSP5 immunoreactivity in NAcore MSNs is more closely associated with cocaine-seeking behavior during reinstatement than with the overall level of drug exposure, supporting a potential role for DUSP5 downregulation in mediating cue-induced relapse mechanisms.

### 3.4. Enhanced Synaptic Plasticity in NAcore MSNs During Cue-Induced Reinstatement

**Figure 4:**
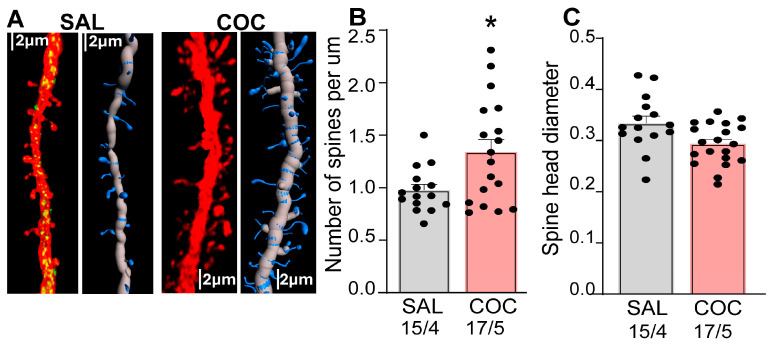
Enhanced synaptic plasticity in NAcore MSNs during cue-induced reinstatement is associated with reduced DUSP5 immunoreactivity. Synaptic plasticity was examined in the same animals analyzed in Figure 2 and Figure 3. (**A**) Representative dendritic segments from the NAcore MSNs in saline- (SAL) and cocaine-treated (COC) rats. The two vertical panels show confocal images of left: DUSP5 puncta (green) and isolated dendrite (red), right: isolate dendrite 3D rendering. (**B**) Increased number of spines per um (spines/µm) in cocaine treated rats compared to saline group (one-way Nested Anova F (1,7) = 7.09, *p* = 0.03). (**C**) No significant difference in spine head diameter between saline- and cocaine-treated rats. Data are shown as mean ± SEM. *N* shown as number of neurons quantified over number of animals in each condition. * *p* < 0.05 comparing COC to SAL.

Previous work by our group and others have shown that cocaine drug-seeking induces structural and functional synaptic plasticity in NAcore MSNs [27,31]. To assess this further, we analyzed dendritic spine morphology in the same dendritic segments used for DUSP5 immunoreactivity quantification (Figure 2), providing a readout of synaptic plasticity (Figure 4). We found a significant increase in spine density (spines per μm) in cocaine-treated animals compared to saline controls (Figure 4B). In contrast, there was no significant difference in spine-head diameter between cocaine- and saline-treated groups (Figure 4C). Taken together, these results show that cocaine exposure enhances dendritic spine density, indicative of structural synaptic plasticity, in NAcore MSNs. Notably, these structural changes occurred in the same dendritic segments that exhibited reduced DUSP5 immunoreactivity, supporting an association between DUSP5 downregulation and maladaptive plasticity linked to cocaine-seeking behavior.

### 3.5. Cocaine Disrupts the Correlation Between DUSP5 Immunoreactivity and Synaptic Plasticity Markers in NAcore MSNs

**Figure 5:**
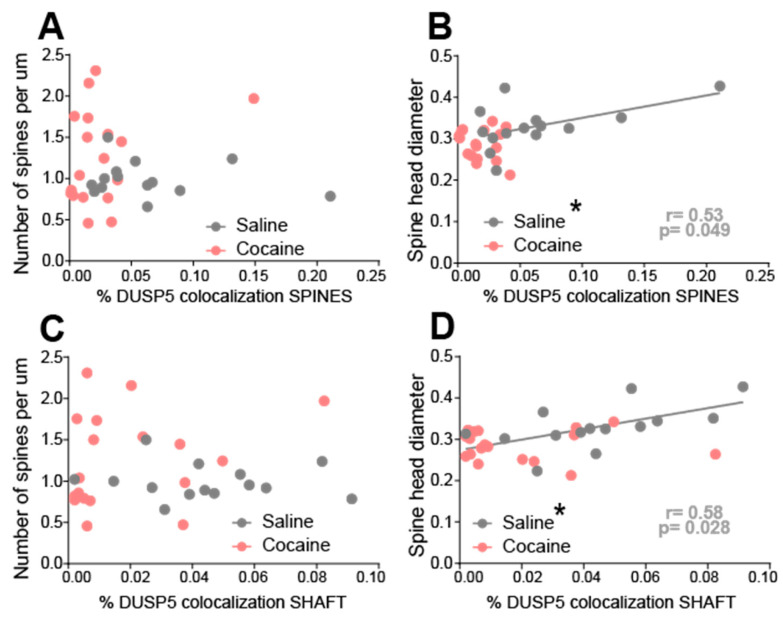
Cocaine-treated rats exhibited a loss of correlation between DUSP5 immunoreactivity in dendritic shafts and spines and synaptic plasticity, as measured by spine head diameter, in NAcore MSNs. Correlations were examined in the same animals analyzed in Figure 2 and Figure 3. (**A**) No significant correlation between DUSP5 immunoreactivity in spine heads and spine density (spines/µm) in either saline- or cocaine-treated groups. (**B**) A significant positive correlation between DUSP5 immunoreactivity in spine heads and spine head diameter was observed in the saline group, but not in the cocaine group (saline: r = 0.53, *p* < 0.05). (**C**) No significant correlation between DUSP5 immunoreactivity in dendritic shafts and spine density in either group. (**D**) A significant positive correlation between DUSP5 immunoreactivity in dendritic shafts and spine head diameter was found in the saline group, but not in the cocaine group (saline: r = 0.58, *p* < 0.05). * *p* < 0.05 positive regression line in saline group.

To determine whether DUSP5 immunoreactivity is associated with structural markers of synaptic plasticity, we performed correlation analyses between DUSP5 signal in dendritic shafts and spines with dendritic spine morphology metrics presented in Figure 4. No significant correlation was observed between DUSP5 immunoreactivity—either in dendritic shafts or spines—and spine density (spines per µm) in either saline- or cocaine-treated groups (Figure 5A,C). In contrast, in saline-treated animals, DUSP5 immunoreactivity in dendritic spines showed a significant positive correlation with spine head diameter(Figure 5B). A similar pattern was observed for DUSP5 in dendritic shafts, where a positive correlation with spine head diameter was present in saline controls but abolished in cocaine-treated rodents (Figure 5D). These findings suggest that under baseline conditions, DUSP5 expression is positively associated with spine head size, potentially reflecting a role in maintaining synaptic integrity. Cocaine exposure appears to disrupt this relationship, indicating a cocaine-induced dysregulation of DUSP5-linked signaling pathways. This disruption may contribute to maladaptive synaptic remodeling in NAcore MSNs, which underlies cocaine-seeking behavior.

## 4. Discussion

The present study identifies DUSP5 as a potential molecular regulator of synaptic plasticity and cocaine-seeking behavior. By integrating behavioral pharmacology, high-resolution imaging of dendritic spine morphology, and transcriptomic mining, we provide convergent evidence that deficiencies in DUSP5 participate in cocaine-induced neuroadaptations.

### 4.1. Dynamic Regulation of DUSP5 Across the Addiction Cycle

Data mining of publicly available single-cell RNA sequencing datasets from rats exposed to i.p. cocaine injections revealed that *DUSP5* mRNA is selectively upregulated in D1-MSN in the NAcore. This transcriptional response was observed 1 h after non-contingent cocaine administration and was not evident for other DUSP family members or in other cell types, indicating a cell-type-specific effect of cocaine on DUSP5 expression. These findings suggest a potential role for DUSP5 in the early molecular adaptations to cocaine exposure and support its involvement in D1-MSN-specific mechanisms within the NAcore [32]. In contrast, using a well-validated model of cocaine SA and cue-induced reinstatement, we found that DUSP5 protein is significantly downregulated in NAcore MSNs following chronic cocaine exposure, as measured 10 days into abstinence during a reinstatement session. Specifically, DUSP5 immunoreactivity was reduced in both dendritic shafts and spines. This discrepancy between mRNA and protein levels may reflect differences in the timing of measurement (1-h post-injection vs. 10 days into abstinence), the mode of cocaine exposure (acute, non-contingent vs. chronic, contingent), or post-transcriptional mechanisms affecting DUSP5. Despite this discrepancy, cocaine clearly alters DUSP5 expression, underscoring its potential role in cocaine-induced neuroadaptations.

### 4.2. DUSP5 Deficiency Correlates with Cocaine-Seeking Behavior

We found that DUSP5 protein levels in dendritic spines and shaft correlated positively with reinstatement behavior, as indexed by active lever presses. Notably, DUSP5 levels did not correlate with total cocaine intake during SA, highlighting its selective involvement in relapse-like behavior rather than cumulative drug exposure. This supports a model in which DUSP5 deficiency contributes to maladaptive plasticity, rather than simply reflecting the pharmacological history of cocaine use.

Furthermore, under control (saline) conditions, DUSP5 levels were positively associated with spine head diameter, a structural correlate of synaptic strength [33,34]. This relationship was abolished following cocaine exposure, indicating that cocaine disrupts the normal association between DUSP5 and synaptic stability. These findings align with previous work showing that cue-induced cocaine reinstatement is associated with elevated spine head diameter [10,26,27,35] or spine density in NAcore MSNs [36,37] and reinforce the hypothesis that DUSP5 acts as a regulator of excitatory synaptic plasticity.

### 4.3. Mechanistic Insights: DUSP5

In this study, we observed DUSP5 protein downregulation after cue-induced reinstatement. Prior work from the same authors, showed that reinstatement engages actin-remodeling processes in NAcore MSNs, particularly via cofilin phosphorylation and spine dynamics, and highlights a central role for D1-MSNs in cue-driven plasticity [27,38,39,40].

As a nuclear ERK1/2 phosphatase, reduced DUSP5 could plausibly influence reinstatement-related signaling, including ERK-dependent regulation of cofilin and its upstream phosphatase Slingshot 1 (SSH1). Additionally, transcriptomic analyses indicated no major transcriptional changes in ERK–cofilin pathway components after cocaine exposure, but this does not establish a functional link between DUSP5 and actin remodeling. Thus, although the ERK–SSH1–cofilin pathway provides a plausible framework through which DUSP5 might regulate structural plasticity during reinstatement, these mechanisms were not directly tested and should be considered hypothesis-generating. Ongoing work in our lab aims to determine whether DUSP5 loss contributes to reinstatement-associated signaling or spine changes, and whether these effects are MSN subtype-specific—critical steps toward clarifying DUSP5’s role in cocaine-induced neuroplasticity.

### 4.4. Limitations of the Interpretation

While our findings offer new insight into the role of DUSP5 in cocaine-induced synaptic plasticity, some limitations should be acknowledged. First, although we observed a robust downregulation of DUSP5 protein in dendritic compartments during cue-induced reinstatement, our study does not directly demonstrate causality between this reduction and the observed structural or behavioral outcomes. Future experiments using targeted DUSP5 overexpression in D1-MSNs will be essential to establish a functional role in relapse-related plasticity.

Second, we rely on correlation analyses to link DUSP5 expression with spine morphology and cocaine-seeking behavior. While the observed dissociation between DUSP5 levels and cumulative cocaine intake supports a specific role in relapse, correlational data cannot rule out the influence of other upstream or parallel mechanisms, including additional phosphatases or kinases.

Third, our model integrates transcriptomic and protein-level data collected at distinct time points and under different cocaine exposure paradigms. Specifically, the snRNA-seq dataset reflects acute, non-contingent cocaine exposure measured 1-h post-injection, while our immunohistochemical analyses were performed after chronic, self-administered cocaine exposure and 10 days of abstinence, followed by reinstatement. This temporal and methodological mismatch may underlie the observed divergence between DUSP5 mRNA upregulation and dendritic protein downregulation, and underscores the need for time-resolved, cell-type-specific measurements of DUSP5 at both the transcript and protein levels throughout the addiction cycle.

Fourth, our interpretation is constrained by the fact that DUSP5 downregulation was assessed specifically during cue-induced reinstatement rather than during abstinence. Reinstatement reflects a relapse-like state driven by conditioned cues in the absence of drug, whereas cocaine intake and abstinence each involve distinct motivational, physiological, and neural processes. Consequently, the molecular alterations observed during relapse-like behavior may not fully capture DUSP5’s role during active drug taking or abstinence. It also remains unclear whether DUSP5 expression fluctuates dynamically across the transition from cocaine use to abstinence and then to cue-induced relapse. Without longitudinal measurements spanning these phases, we cannot determine whether reduced DUSP5 levels during reinstatement arise from abstinence-related adaptations or from cue-evoked neuronal activation. Nevertheless, the observed correlation between active lever responding and reduced synaptic stability as DUSP5 decreases suggests a potential functional role for DUSP5 in relapse vulnerability.

Finally, while our data suggest that post-translational regulation via ERK–SSH1–cofilin signaling is a likely mechanism linking DUSP5 downregulation to altered spine dynamics, we did not directly measure ERK or p-cofilin levels in the same neurons analyzed for DUSP5. Future studies should integrate multiplexed imaging or biochemical approaches to dissect the spatial and temporal coordination of these molecular changes within D1-MSNs. These limitations highlight the need for further mechanistic work to validate DUSP5 as a key modulator of cocaine-induced neuroplasticity and to determine its therapeutic potential in relapse prevention.

## 5. Conclusions

Using a well-validated model of cocaine SA and cue-induced reinstatement, we observed a striking downregulation of DUSP5 protein in dendritic spines and shafts of NAcore MSNs, and single-nucleus transcriptomic mined data showed selective upregulation of *DUSP5* mRNA in D1-MSNs following acute cocaine exposure. This transcription–protein dissociation suggests complex, phase-specific regulation of DUSP5 across the addiction cycle. Importantly, DUSP5 protein levels did not correlate with total cocaine intake but instead showed a strong association with cue-induced reinstatement behavior, reinforcing its role in relapse vulnerability rather than in cumulative drug exposure. Furthermore, cocaine disrupted the normal correlation between DUSP5 and spine head diameter and spine density, a structural hallmark of synaptic potentiation, implicating DUSP5 loss in maladaptive plasticity mechanisms. Overall, this work highlights DUSP5 as a promising molecular target for interventions aimed at disrupting the synaptic plasticity that underlies cocaine use.

## Figures and Tables

**Table 1:** Stats corresponding to Figure 1.

Gene	Condition	*p*-Value	*p*-adj	log2 Fold Change	Significant?
Dusp5	Acute cocaine	<0.00001	0.0003	2.778	Yes
	Repeated cocaine	0.509	0.997	0.416	No
Dusp6	Acute cocaine	0.001	N/A	2.837	No
	Repeated cocaine	0.747	0.997	−0.303	No
Dusp7	Acute cocaine	0.093	0.961	−0.908	No
	Repeated cocaine	0.998	0.997	0.006	No
Dusp14	Acute cocaine	0.018	0.474	0.758	No
	Repeated cocaine	0.495	0.997	0.181	No
Dusp15	Acute cocaine	0.819	0.983	−0.115	No
	Repeated cocaine	0.766	0.997	0.123	No
Limk1	Acute cocaine	0.742	0.983	−0.162	No
	Repeated cocaine	0.741	0.997	0.179	No
Limk2	Acute cocaine	0.278	0.983	−0.271	No
	Repeated cocaine	0.572	0.997	−0.125	No
Mapk1	Acute cocaine	0.741	0.983	−0.044	No
	Repeated cocaine	0.609	0.997	−0.059	No

## Data Availability

The original contributions presented in this study are included in the article material. Further inquiries can be directed to the corresponding authors.

## Supplementary Materials

The following supporting information can be downloaded at: https://www.mdpi.com/article/10.3390/cells15010032/s1, Supplemental Figure S1. Quantifying dendritic DUSP5 protein immunoreactivity in labeled MSNs from a saline-treated control animal. This figure presents the rendering and quantification of a confocal image showing a virus-labeled dendritic segment from a medium spiny neuron (MSN). Panel 1 illustrates the dendritic structure filled using the filament module in the IMARIS software, with spine heads color-coded based on their diameters. Panel 2 displays a 3D-flattened, masked representation of the dendritic shaft and spines (in transparent white), overlaid with the surrounding DUSP5 signal (green). Panel 3 isolates the 3D-flattened, masked dendritic shaft with the adjacent DUSP5 signal, while Panel 4 focuses on the 3D-flattened, masked dendritic spines and their surrounding DUSP5 signal. Supplemental Figure S2. DUSP5 mRNA is selectively upregulated in D1-MSNs of the NAc following acute cocaine exposure. Top: Schematic of the experimental design from a publicly available single-nucleus RNA-sequencing (snRNA-seq) dataset [29]. Rats were treated with either acute (1×) or repeated (7×) intraperitoneal (i.p.) injections of cocaine or saline. One hour after the final injection, ~2 mm tissue punches from the NAc were collected for snRNA-seq using the 10× Genomics platform. The integrated dataset contains transcriptomic profiles from 39,254 nuclei, with data stratified by cell type. Bottom: Gene expression levels (log-transformed) for Dusp5, Dusp6, Dusp7, Dusp14, Dusp15, and Dusp27 across distinct brain cell populations, including Drd1- and Drd2-expressing MSN, interneurons, glial cells, and astrocytes. Pink lines represent expression following cocaine, and teal lines represent saline controls. Notably, Dusp5 mRNA is selectively upregulated in Drd1-MSNs (D1-MSNs) following acute cocaine administration, while other DUSPs show no consistent cocaine-related changes across cell types. This Dusp5-specific induction in D1-MSNs highlights its potential role in mediating early, cell type–specific transcriptional responses to cocaine exposure in the NAc. Supplemental Figure S3. (A) Time course of active and inactive lever pressing of SA and extinction in saline and cocaine- and saline-treated rats used for DUSP5 immunoreactivity. [Coc SA: two-way ANOVA repeated measures over time, time *F*_(9,68)_ = 0.88, *p* = 0.54; lever presses *F*_(1,8)_ = 5.35, *p* = 0.04; interaction *F*_(9,68)_ = 0.708, *p* = 0.70] [Coc Ext: two-way ANOVA repeated measures over time, time *F*_(7,56)_ = 22.42, *p* < 0.001; lever presses *F*_(1,8)_ = 1.247, *p* = 0.296; interaction *F*_(7,56)_ = 5.350, *p* < 0.001]. [Sal SA: two-way ANOVA repeated measures over time, time *F*_(9,54)_ = 0.805, *p* = 0.613; lever presses *F*_(1,6)_ = 0.086, *p* = 0.778; interaction *F*_(9,54)_ = 0.498, *p* = 0.868] [Sal Ext: two-way ANOVA repeated measures over time, time *F*_(7,42)_ = 3.388, *p* < 0.005; lever presses *F*_(1,6)_ = 0.301, *p* = 0.603; interaction *F*_(7,42)_ = 0.451, *p* = 0.864]. (B) Total number of infusions for saline and cocaine-treated rats. (C) Number of active and inactive lever presses during cue-induced reinstatement. [two-way ANOVA, treatment *F*_(1,14)_ = 4.011, *p* = 0.065; active vs inactive *F*_(1,14)_ = 12.97, *p* = 0.003; interaction *F*_(1,14)_ = 6.96, *p* = 195]. Data are shown as mean ± SEM. *N* corresponds to 5 cocaine animals and 4 saline animals. ALP= active lever presses, ILP= inactive lever presses.
