# Supplementary material for: DUSP5 Downregulation in Nucleus Accumbens Core Correlates with Cocaine-Induced Maladaptive Synaptic Plasticity
Source: Cells. 2025 Dec 23;15(1):32. doi: 10.3390/cells15010032 (PMC12785552; doi:10.3390/cells15010032)
Supplement: Supplementary file 1 [file cells-15-00032-s001.zip › cells-3919990-supplementary.pdf]

Supplemental files

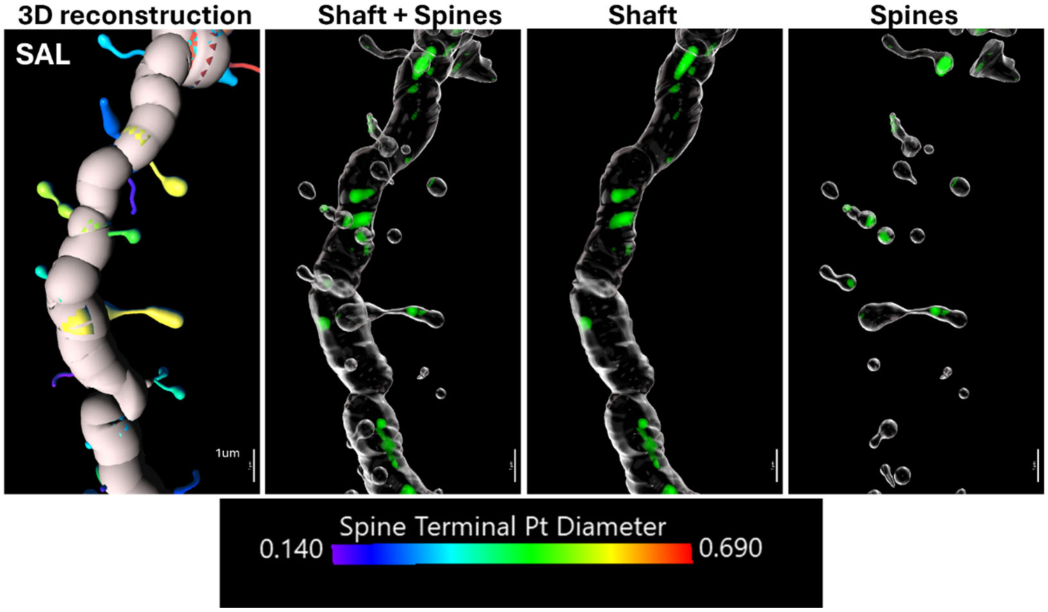

Supplemental Figure S1. Quantifying dendritic DUSP5 protein immunoreactivity in labeled MSNs from a saline-treated control animal. This figure presents the rendering and quantification of a confocal image showing a virus-labeled dendritic segment from a medium spiny neuron (MSN). **Panel 1** illustrates the dendritic structure filled using the filament module in the IMARIS software, with spine heads color-coded based on their diameters. **Panel 2** displays a 3D-flattened, masked representation of the dendritic shaft and spines (in transparent white), overlaid with the surrounding DUSP5 signal (green). **Panel 3** isolates the 3D-flattened, masked dendritic shaft with the adjacent DUSP5 signal, while **Panel 4** focuses on the 3D-flattened, masked dendritic spines and their surrounding DUSP5 signal.

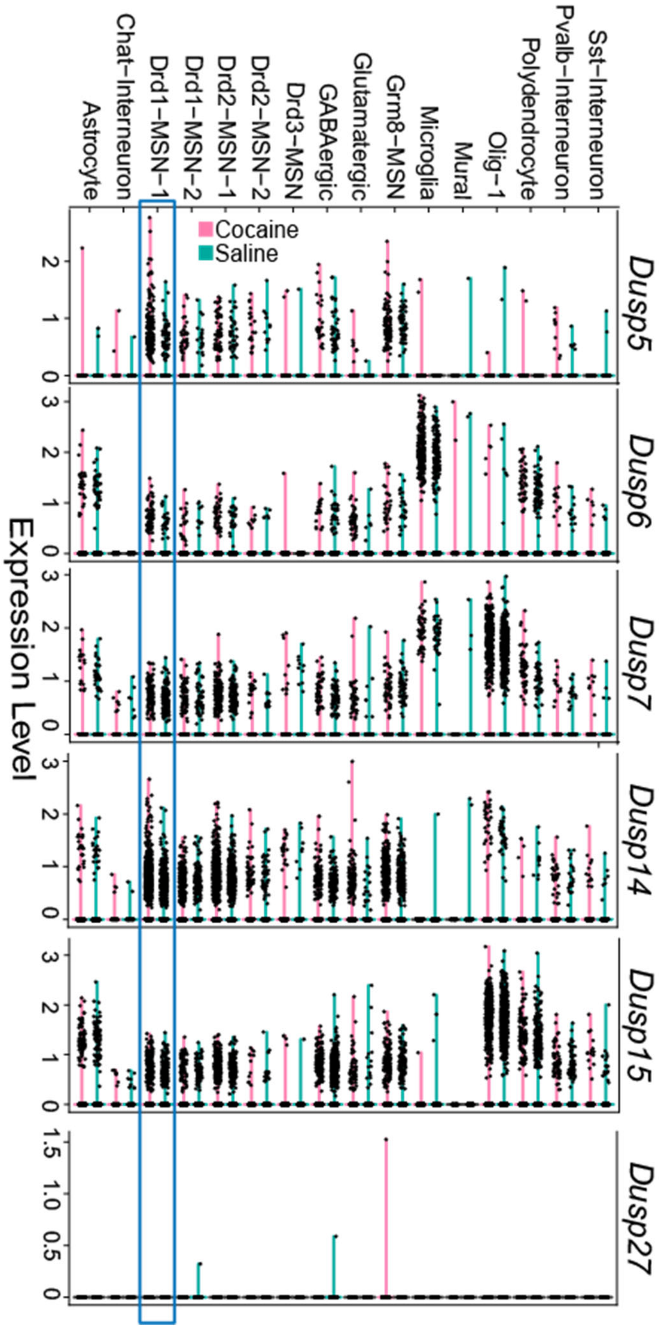

Supplemental Figure S2. DUSP5 mRNA is selectively upregulated in D1-MSNs of the NAc following acute cocaine exposure. **Top:** Schematic of the experimental design from a publicly available single-nucleus RNA-sequencing (snRNA-seq) dataset [29]. Rats were treated with either acute (1x) or repeated (7x) intraperitoneal (i.p.) injections of cocaine or saline. One hour after the final injection, ~2 mm tissue punches from the NAc were collected for snRNA-seq using the 10x Genomics platform. The integrated dataset contains transcriptomic profiles from 39,254 nuclei, with data stratified by cell type. **Bottom:** Gene expression levels (log-transformed) for Dusp5, Dusp6, Dusp7, Dusp14, Dusp15, and Dusp27 across distinct brain cell populations, including Drd1- and Drd2-expressing MSN, interneurons, glial cells, and astrocytes. Pink lines represent expression following cocaine, and teal lines represent saline controls. Notably, Dusp5 mRNA is selectively upregulated in Drd1-MSNs (D1-MSNs) following acute cocaine administration, while other DUSPs show no consistent cocaine-related changes across cell types. This Dusp5-specific induction in D1-MSNs highlights its potential role in mediating early, cell type–specific transcriptional responses to cocaine exposure in the NAc.

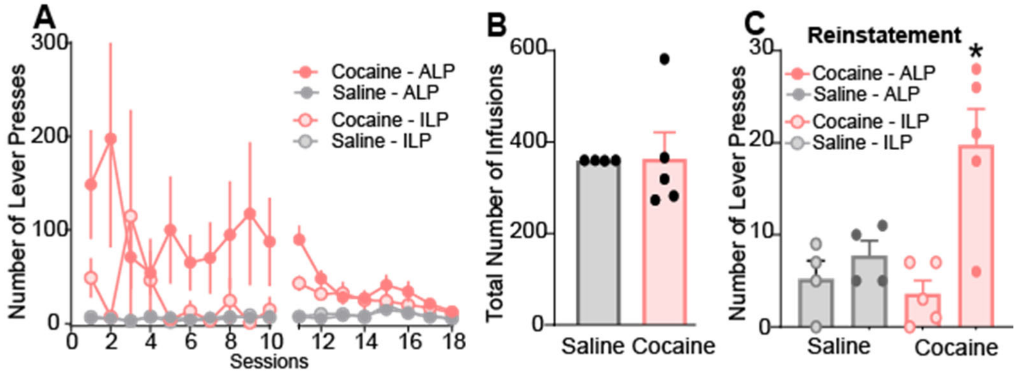

Supplemental Figure S3. A) Time course of active and inactive lever pressing of SA and extinction in saline and cocaine- and saline-treated rats used for DUSP5 immunoreactivity. [Coc SA: two-way ANOVA repeated measures over time, time $F_{(9,68)}$= 0.88, p=0.54; lever presses $F_{(1,8)}$ = 5.35, p = 0.04; interaction $F_{(9,68)}$ = 0.708, p = 0.70] [Coc Ext: two-way ANOVA repeated measures over time, time $F_{(7,56)}$= 22.42, p<0.001; lever presses $F_{(1,8)}$ = 1.247, p = 0.296; interaction $F_{(7,56)}$ = 5.350, p<0.001]. [Sal SA: two-way ANOVA repeated measures over time, time $F_{(9,54)}$= 0.805, p=0.613; lever presses $F_{(1,6)}$ = 0.086, p = 0.778; interaction $F_{(9,54)}$= 0.498, p=0.868] [Sal Ext: two-way ANOVA repeated measures over time, time $F_{(7,42)}$= 3.388, p<0.005; lever presses $F_{(1,6)}$ = 0.301, p = 0.603; interaction $F_{(7,42)}$ = 0.451, p=0.864]. **B)** Total number of infusions for saline and cocaine-treated rats. **C)** Number of active and inactive lever presses during cue-induced reinstatement. [two-way ANOVA, treatment $F_{(1,14)}$= 4.011, p = 0.065; active vs inactive $F_{(1,14)}$ = 12.97, p = 0.003; interaction $F_{(1,14)}$ = 6.96, p = 195].

Data are shown as mean ± SEM. *N* corresponds to 5 cocaine animals and 4 saline animals. ALP= active lever presses, ILP= inactive lever presses.
